# COVID-19 and its impact on mental health as a function of gender, age, and income

**DOI:** 10.1007/s44192-022-00025-y

**Published:** 2023-01-23

**Authors:** Namrata Gulati, Chandni Nanda, Ramandeep Kaur Hora

**Affiliations:** 1 Faculty of Economics, South Asian University, Akbar Bhawan, Satya Marg, Chanakyapuri, New Delhi Delhi, 110021 India; 2 Digital Assets & Outreach Team at Zigram Data Technologies Pvt. Ltd, Delhi, India

**Keywords:** COVID-19, Anxiety score, Ordered logit model, Principal factor analysis, Regression analysis

## Abstract

This paper examines the impact of COVID-19 on the mental health of people from different socioeconomic classes. This has been done by creating an anxiety score, based on responses to a set of questions that were asked as part of a two-round telephonic survey done by Young Lives Data for India. Using this index, anxiety levels have been classified as high, medium, and low. As the dependent variable has an ordered nature, an ordered logit model has been used for regression. According to the results, job loss, death of the family’s earning member or mishap in the family, and price increases all contributed to increased anxiety. The analysis indicates that anxiety levels among women were higher than among men because of the increased burden of household chores and childcare responsibilities. Also, anxiety levels were higher among those who perceived themselves as rich or poor as opposed to those who were comfortable in their income group. There was a higher anxiety level among the rich due to income loss and increased household responsibilities. A high percentage of children and elders reported feeling anxious; school closures and a lack of social interaction caused stress. Isolation also plagued the elderly. Furthermore, many students couldn’t avail online learning opportunities due to a lack of resources. The government implemented many policies to mitigate these issues, which included those to mitigate the immediate hunger problem.

## Introduction

The global pandemic of COVID-19 had afflicted several nations in a short period of time. It was India’s worst health crisis since Independence [19]. To contain the spread of COVID-19, the Government of India declared the disease a national disaster on March 25, 2020. The longest lockdown in history ensued after being extended more than three times. During the lockdown, education institutions were shut down, and flights, trains, metros, and large gatherings of any kind were prohibited. Only essential services were allowed to operate. This situation persisted for a long in various forms and varying degrees across states. As a result of the pandemic and the prolonged lockdown, health, economy, livelihood, and social and professional interactions all suffered. Millions of people lost their livelihood, and many migrant workers were forced to return to their villages [24]. The effects of all this have been dramatic in many ways.

Due to the pandemic and associated factors, a range of emotions were triggered, resulting in an overall decline in well-being. Whether it was the loss of a job, a death in the family, or an increase in household responsibilities, all lead to a decline in mental health. In a 2020 telephonic survey conducted by Young Lives Data in the Indian states of Andhra Pradesh and Telangana, 89% said they felt nervous about COVID-19-related circumstances. There was, however, a wide variation in the extent of the effects on mental health. The reason for this was primarily due to the huge heterogeneity of castes, statuses, and income levels in India. In India, heterogeneity of class and caste impacts a wide range of socioeconomic outcomes. This has been confirmed by many authors, including [16, 18, 20, 33]. This resulted in some individuals being better able to cope with the shock, while others were relatively less resilient.

The depth of an emotional wound can be determined by the mental state of a person. This paper aims to empirically assess the effects of COVID-19 on mental health. Specifically, the aim is to determine whether COVID-19 had a differential effect on individuals belonging to different socioeconomic groups, what factors caused the situation to worsen for one group compared with another, and why were some people better able to resist pressure than others. To understand these, it is first necessary to clearly define mental health. Thus, an anxiety index was developed to achieve this. Using this anxiety index, three categories of anxiety levels were created—high, medium, and low. The purpose of this study was to determine how likely it was for individuals from a particular socioeconomic group to experience a given level of anxiety.

A few measures were taken by the state governments to mitigate the effects of COVID-19. These measures were designed to ease economic hardship and ensure food security. In most cases, the respondents benefitted from one or more of the government’s projects. We also aim to evaluate how effective were these policy measures in ameliorating an individual’s situation.

This analysis was conducted using data from two rounds of telephonic surveys conducted by Young Lives Data in 2020 in the Indian states of Andhra Pradesh and Telangana. More details about the data can be found in Sect. 2.

This paper is organized as follows: Sect. 2 discusses data. It also includes a discussion of the variables and descriptive statistics. The methodology and regression results are presented in Sect. 4. The discussion on policy measures is presented in Sect. 5, followed by a conclusion in Sect. 6.

## Data and variables

The study draws its data from the first and second calls of the “Listening to Young Lives at Work: COVID-19 Phone Survey” for Andhra Pradesh and Telangana. A Young Lives Phone Survey was conducted to assess the effects of COVID-19 on young people’s health and general well-being in the short and medium term. For this purpose, Young Lives’ main longitudinal sample was used. The main survey of Young Lives examines the changing nature of childhood poverty in four developing countries—Ethiopia, India, Peru, and Vietnam. Approximately 12,000 children and families are surveyed every three to 4 years by Young Lives. A survey was first conducted in 2002, and a more recent survey was done in 2016. It includes children born in 2001–2002 and those born in 1994–1995.

Listening to Young Lives at Work: COVID-19 Phone Survey is an adaptation of Round 6 of the longitudinal Young lives survey. After the COVID-19 crisis, fieldwork for the longitudinal Young lives survey was halted and, instead, a three-part telephone survey was conducted to assess the effects of the crisis. Having developed a long-term relationship with participants, the team was able to obtain up-to-date contact information for 90% of 2016 respondents.

The sample for this study is based on data collected by Young Lives India since 2002 in the state of undivided Andhra Pradesh. On June 2, 2014, Andhra Pradesh was divided into Telangana and Andhra Pradesh. After the partition of Andhra Pradesh in 2014, the Young Lives sample was spread across three districts in Andhra Pradesh (Srikakulam, West Godavari, and Cudappah) and four districts in Telangana (Karimnagar, Mahbubnagar, Anantapur, and Hyderabad). A multistage, purposeful, and random sampling approach was used to select the sample. A total of 15 rural sites were sampled in the state, along with 5 urban sites (including the capital, Hyderabad). Within each sentinel site, 100 households with children born in 2001–2002 and 50 households with children born in 1994–1995 were randomly selected, while the sites themselves were identified based on predetermined criteria. Refer to Technical Note 2 [26] for more information on sampling rigour. Being pro-poor, the sample is only broadly representative of the state [26], and it is not representative of India at large.

The data collection method used for “Listening to Young Lives at Work: COVID-19 Phone Survey” was a telephone interview with computer-assisted transcription (CATI). During the first survey in 2020, 887 households in the older cohort and 1,863 households in the younger cohort were surveyed. The survey took place between June and July 2020.

In the second call conducted between September and October 2020, 886 households were surveyed in the younger cohort, while 1,868 households were surveyed in the older cohort. The observation was made at the individual and family/household levels. The team tracked 98.2% of the younger cohort after the first call and 97% of the older cohort after the second call. In both surveys, consent of the participants was requested during the initial conversation. This required the enumerators to sign a declaration that the data had been collected after explaining everything related to the survey to the participants, disclosing to them the consent text, and answering all their questions and queries. Additionally, enumerators were required to sign a statement agreeing to regulate their behaviour according to the Young Lives Code of Conduct.

## Variables and descriptive statistics

To measure the anxiety level of the respondent, an anxiety score was created using the data discussed above. The “anxiety score” is based on the interviewees’ responses (0 = No, not at all; 1 = Yes, even if a little bit) to the following seven questions:

- Feeling nervous, anxious, or on edge
- Not being able to stop or control worrying
- Worrying too much about different things
- Trouble relaxing/can’t relax
- Being so restless that it’s hard to sit still
- Becoming easily annoyed or irritable
- Afraid that something awful might happen

This anxiety measure is comparable to the Generalised Anxiety Disorder Assessment (GAD-7) index [25, 38] in that it is based on the same set of questions. However, instead of calculating frequency by the number of days, answers were evaluated by No or Yes. The numerical values 0 and 1 were assigned to No and Yes, respectively, and the responses to all questions were added. Exploratory Factor Analysis (EFA) was used to first determine the factors which had to be retained to construct the score which was then constructed by using two methods—the EFA and simple addition. EFA traces its roots back to Spearman (1904) given [39]. EFA is based on the concept that unobserved variables can explain the variation in scores for observed variables [10]. In this method. it is assumed that a correlation coefficient between two variables is due to the common cause of both variables, which is a third variable. The correlation between the two indices is 0.94.

Anxiety scores ranged from 0 to 8. For the analysis outcome variable, anxiety scores have been divided into three broad categories as follows:

- The anxiety score of 0 or 1 has been coded as “low anxiety.”
- The anxiety score between 2 and 4 is coded as “medium anxiety.”
- The anxiety score between 5 and 7 is coded as “high anxiety.”

Table 1 shows the percentage distribution of different variables across three levels of anxiety—low, medium, and high. A relatively higher level of anxiety was reported by women, young people, and those households which identified themselves as poor or struggling. A family member suffering from COVID-19 also added to the anxiety and nervousness.

**Table 1:** Distribution of anxiety with respect to key variables

	Low anxiety	Medium anxiety	High anxiety	Total
Gender of Household Member				
Male	60.18	29.16	10.66	49.42
Female	57.98	29.87	12.15	50.58
Households (HH) Perception of its Type before Covid out-break				
Rich	62.81	28.42	8.78	4.01
Comfortable	57.66	31.47	10.88	72.95
Struggling	54.73	31.17	14.1	15.27
Poor	48.05	36.3	15.65	7.77
Cohort				
Older cohort	57.44	32.67	9.89	41.8
Younger cohort	56.1	30.97	12.93	58.2
Rural or Urban indicator				
Rural	62.3	30.1	7.6	50.75
Urban	50.82	33.31	15.87	49.25
Rise in prices of major food items				
No	67.19	26.8	6.01	41.32
Yes	49.25	35.12	15.64	58.68
Relation with father as a result of Covid				
It has deteriorated	15.77	45.75	38.48	7.15
It remains the same	37.65	39.15	23.2	46.72
It has improved	29.32	39.97	30.71	46.13
Relation with mother as a result of Covid				
It has deteriorated	13.45	42.05	44.51	4.22
It remains the same	37.91	37.15	24.94	38.72
It has improved	29.79	41.78	28.43	57.06
Job loss				
No Job Loss	63.39	28.74	7.87	62.32
Yes Job Loss	45.51	36.55	17.94	37.68
Currently have job				
No	47.21	35.98	16.81	83.08
Yes	51.47	39.74	8.78	16.92
Temporarily laid off				
Yes laid off	46.43	41.51	12.06	67.49
Not Laid off	61.94	36.08	1.98	32.51
Work during lockdown				
Could Work	54.86	34.06	11.08	50.96
Could Not Work	46.74	37.55	15.71	27.03
No Job During lockdown	53.52	32.52	13.96	22.01
Mishap earning member of HH				
No Mishap	59.51	30.85	9.63	90.6
Yes Mishap	29.14	39.63	31.23	9.4
Spent more time on HH chores				
Agree	53.67	32.31	14.03	60.08
Partially agree	58.82	31.58	9.6	19.73
Disagree	63.51	29.89	6.6	20.19
Spent more time on childcare				
Agree	54.27	32.8	12.94	42.24
Partially agree	57.77	29.55	12.68	11.1
Disagree	58.58	31.17	10.25	46.66
Anyone got Covid				
No Covid	57.88	31.28	10.84	95.34
Yes Covid in home	31.34	39.95	28.71	4.66
Total	56.67	31.68	11.66	100

Compared with those living in urban neighbourhoods, Indians living in rural neighbourhoods reported less anxiety. It could be due to the fact that the price increase in the urban areas was steeper than in the poor ones. It is also likely that the individuals felt more at ease in the rural neighbourhood because it has a culture of sharing and helping each other in times of distress.

Furthermore, anxiety levels increased when a family member lost their job or an individual was unable to work during the lockdown. All of these factors made a person more financially vulnerable, making them more paranoid.

Table 1 shows that around 13% of the younger cohort reported high anxiety levels. Many factors could have contributed to high anxiety. Because of the closure of the physical school, and also due to the fact that many of them lacked mobile phones or laptops to attend online classes, many of them had to discontinue their studies. During school closures, many students were unable to take advantage of the midday meal program implemented in government schools, thus aggravating hunger problems during the lockdown.

## Methodology and regression results

The regression equation used to examine the influence of different factors on the anxiety of an individual was

1 $$anxiety_{i}^{*} = \alpha + \beta_{j} \sum\limits_{j = 1}^{N} {X_{k} } + \varepsilon_{i}$$

where $\beta$ is a vector of coefficients, ${X}_{k}$ is the vector of independent variables such as age, gender and household type, and their interaction with factors leading to anxiety, and ${\varepsilon }_{i}$ is the error term, distributed normally along the observations $.$ The dependent variable in the model captures an individual’s perception of his/her anxiety level because of COVID-19 and related issues. This model has been estimated using the simple Ordinary Least Squares (OLS) method.

As discussed, the anxiety index has been divided into three ordered categories—low, medium, and high, to get a clearer understanding of how the intensity of anxiety changes from one category to the other. Theoretically, division of the model into three categories can be presented in the following way:

$${\mathrm{anxiety}}_{i}=\mathrm{low}=1 \mathrm{if} 0<{\mathrm{anxiety}}_{i}^{*}\le {\tau }_{1;}$$

$${\mathrm{anxiety}}_{i}=\mathrm{medium}= 2 \mathrm{if} {\tau }_{1}<{\mathrm{anxiety}}_{i}^{*}\le {\tau }_{2;}$$

$${\mathrm{anxiety}}_{i}=\mathrm{high}= 3 \mathrm{if} {\tau }_{2}<{\mathrm{anxiety}}_{i}^{*}\le {\tau }_{3;}$$

where ${\tau }_{1 ,}{\tau }_{2 },$ and ${\tau }_{3}$ are cut-off values.

The different levels of ${\mathrm{anxiety}}_{i}$ have been written as a function of the continuous variables ${\mathrm{anxiety}}_{i}^{*},$ which can be observed from the data.

In this model, the probability for any given outcome category (*m* = 1, 2, 3) is

2 $$\Pr \left( {y = \left. m \right|x} \right) = \left\{ \begin{gathered} F\left( {\tau_{1} - x\beta } \right)\,m = 1, \hfill \\ F\left( {\tau_{2} - x\beta } \right)\,F\left( {\tau_{1} - x\beta } \right)\,m = 2, \hfill \\ 1 - F\left( {\tau_{2} - x\beta } \right)\,m = 3\, \hfill \\ \end{gathered} \right.\,$$

Hhere *F* is the logistic cumulative density function, *τ* is a cut-off point, $x$ is a vector of independent variables such as gender, income, age, etc., *β* is a vector of logit coefficients that does not vary across equations, and *m* is the category and its corresponding logit equation (Long 1997: 121–22 [27]; Powers & Xie 2000: 212 [36]). The model is estimated using the maximum likelihood method.

The results from the OLS and OLOGIT regression are given in Table 2. The level of anxiety is used as the dependent variable in the OLS model, whereas the ordered category of anxiety serves as the dependent variable in the OLOGIT mode. Column 1 reports the results from the simple OLS regression. *β* coefficient from the OLOGIT regression is given in column 2. Odds ratio is listed in Column 3. The odds ratios for the restricted regression based on Female, Rich, and Comfortable households are given in columns 4, 5, and 6, respectively.

**Table 2:** Regression results

Variables 1	Coding 2	OlS Coefficient 3	OLOGIT Coefficient 4	Odds ratio 5	Odds ratio (Female) 6	Odds ratio (Poor) 7	Odds ratio (Rich) 8
Household deems itself Struggling or poor before covid	1 if the answer is yes, 0 otherwise	0.029**	0.091***	1.095***	1.131***		
		−0.0112	−0.0327	−0.0358	(0.0518)		
Household deems itself rich before covid	1 if the answer is yes, 0 otherwise	0.085***	0.281***	1.324***	1.302**		
		−0.0274	−0.0762	−0.1009	(0.1407)		
Suffered from job loss	1 if respondent agrees, 0 otherwise	0.207***	0.579***	1.784***	1.747***	1.491***	3.599***
		−0.0095	−0.0274	−0.049	(0.0669)	(0.0849)	(0.6184)
Spent more time on household chores than before	1 if respondent agrees, 0 otherwise	0.104***	0.346***	1.413***	1.443***	1.554***	2.333***
		−0.0123	−0.0379	−0.0536	(0.0780)	(0.1205)	(0.4862)
Increase in price of major food items consumed	1 if respondent agrees, 0 otherwise	0.267***	0.777***	2.175***	2.123***	1.600***	1.232
		−0.0095	−0.0284	−0.0617	(0.0840)	(0.1012)	(0.2075)
Illness, injury, or death of income earning member of household	1 if the answer is yes, 0 otherwise	0.338***	0.853***	2.347***	2.385***	2.670***	2.963***
		−0.0148	−0.0416	−0.0978	(0.1398)	(0.2143)	(0.6180)
Spent more time taking care of children than before	1 if respondent agrees, 0 otherwise	0.068***	0.184***	1.202***	1.144***	0.984	1.584***
		−0.0096	−0.0283	−0.034	(0.0450)	(0.0621)	(0.2587)
Gender	1 if female, 0 if male	0.043***	0.117***	1.124***		1.184***	1.101
		−0.0089	−0.0262	−0.0295		(0.0667)	(0.1754)
Age	continuous variable	−0.001***	−0.003***	0.997***	0.996***	0.995***	1.002
		−0.0002	−0.0007	−0.0007	(0.0009)	(0.0014)	(0.0040)
In cash Enter in currency unit (in '000 VND)					0.999**	1.000	0.999
					(0.0003)	(0.0004)	(0.0012)
/cut1			1.231***	3.425***	2.917***	2.362***	4.295***
			−0.0459	−0.1573	(0.1839)	(0.2156)	(1.1901)
/cut2			3.071***	21.565***	17.828***	13.621***	60.186***
			−0.05	−1.0784	(1.2180)	(1.3455)	(19.3983)
Constant		1.231***					
		−0.0148					
Observations		23,117	23,117	23,117	11,736	4,709	630
Standard errors in parentheses	*** p < 0.01, ** p < 0.05, * p < 0.1						

*p < 0.1, **p < 0.05, ***p < 0.01

There are some aspects that need to be qualified before interpreting the results. For simplicity, the poor and struggling categories were grouped together in regressions. The probability of the highest anxiety level increases (decreases) when a parameter *β* from the OLOGIT regression is positive (negative) as the value of the variable *X* increases, while the probability of the lowest anxiety level decreases (increases).

Similarly, if the odds ratio is greater than 1, it implies that the likelihood of higher anxiety captured by the latent variable ${\mathrm{anxiety}}_{i}^{*}$ increases, but if the odds ratio is less than 1 then it falls. The explanatory variables whose *β* coefficient is positive and the odds ratio is greater than 1 are: spending more time on household chores or children than before, being sick or injured, losing a job, or experiencing an increase in the prices of major food items.

One can rank the influence of each variable on anxiety by comparing the estimated coefficients of all the variables mentioned above. Table 2 shows that the largest positive parameter is the one associated with illness, injury, or death of a household income-earner, followed by job loss. This meant that financial shock contributed significantly to anxiety. The increase in food prices was also a major factor in increasing anxiety, while spending more time on child care and other household chores had a positive but a relatively smaller impact.

It is worth noting that households which perceived themselves as wealthy had higher anxiety than households that perceived themselves as comfortable. In addition, households that believed themselves to be poor or struggling before COVID-19 had a positive coefficient, indicating that they were also more stressed than those who believed themselves to be comfortable. The coefficient for rich households (*β* = 0.28) is greater than the coefficient for poor or comfortable households (*β* = 0.091) indicating that rich households were more anxious. Odds ratios convey the same conclusions as the *β* coefficients. Compared to people belonging to the comfortable income category, the odds of reporting high anxiety are 9% higher for the poor or struggling income group, and 32.4% higher for the rich.

It is worth exploring the reasons for the same in more detail. Based on the fifth column of the Table 2, it is evident that the odds ratio for job loss for rich households is 3.59. This is significantly higher than for poor or comfortable households indicating that job loss for relatively rich households had a very strong bearing on their level of anxiety. Perhaps this is caused by the limited chances of finding a similar job by the rich after losing one. In a similar manner, a mishap occurring to the earning member of a richer household led to higher anxiety than it did for a poorer or less comfortable household. Moreover, since the rich in India generally outsource household chores, spending more time on them became more challenging, resulting in higher anxiety levels.

**Fig. 1:**
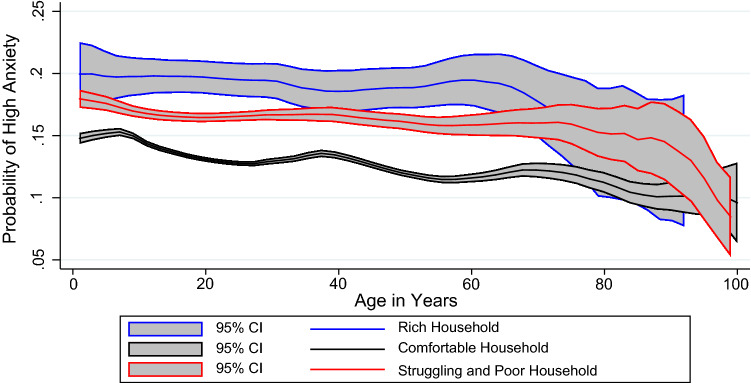
…

Figure 1 depicts the change in probability of high anxiety for different income groups with respect to age. The graph is in line with the results. Anxiety levels were highest among rich households, followed by poor and comfortable households. The reasons for this have already been discussed.

The variable gender was a positive parameter with an odds ratio greater than 1, indicating that women experienced more anxiety than men on an average. Loss in family’s financial stability was critical to women’s anxiety be it because of their own job loss or a mishap with the earning member of the family or because of the increase in income, as is also clear from column 4. Also, women experienced higher anxiety because they were now more engaged in child care and household responsibilities than men.

## Policy measures and their impact

Because of the lockdown, Indian workforce shrank by 122 million in April 2020 despite government encouragement not to lay off workers or reduce wages. The closure of factories and workplaces resulted in the loss of thousands of jobs for migrant workers. As per the report of Young Lives Data, although many households in India were exposed to COVID-19, the economic effects of lockdown policies have been more significant than the health impacts.

In the midst of the pandemic, food security had become a major concern due to the loss of jobs and the rise in food prices. At some point after the outbreak, about 16% of respondents reported that they ran out of food due to lack of money or other resources. One in six Young Lives households in Peru, India, and Ethiopia reported running out of food at some point since the beginning of the crisis. This was especially true for households that identified themselves as poor and food-insecure earlier in 2016. This situation resulted in high levels of anxiety​. More than 90% of the young people in the states of Andhra Pradesh and Telangana in India, said that they felt nervous about the situation.

The government took several measures to ease migrants’ plight, to alleviate their economic losses, and especially to address food security issues. A few of these measures and their affects in ameliorating the stress level have been listed in the Table 3. Among the sampled households, the majority, especially those from the most vulnerable backgrounds, received some kind of government assistance. In Telangana State, 90% of households received certain form of government assistance, while in Andhra Pradesh, 93% responded that they received assistance. Support in both states was primarily provided through direct cash transfers, transfers to Jandhan accounts, and food rations, although in many cases the support consisted of a small basket of food or face masks. The following table summarizes the impact of government interventions on reducing anxiety either by alleviating hunger directly through food transfer or indirectly through income assistance.

**Table 3:** Policies measures to ameliorate conditions of people

	Low level of anxiety	Medium level of anxiety	High level of anxiety	Total
Wheat 1 kg per family				
No wheat	88.66	79.15	95.31	86.55
Yes Wheat	11.34	20.85	4.69	13.45
Sugar half kg per family				
No Sugar	22.39	21.04	7.81	21.52
Yes Sugar	77.61	78.96	92.19	78.48
Face masks 3 per head				
No Masks	25.81	18.58	4.69	23.27
Yes Masks	74.19	81.42	95.31	76.73
*Jandhan* A/c holder 500 pm for 3 months				
No Jandhan500	71.85	65	69.53	69.95
Yes Jandhan500	28.15	35	30.47	30.05
Food bag mid-day meal takers				
No mid-day meal	100	100	84.38	99.53
Yes mid-day meal	0	0	15.63	.47
Subsidised fruits vegetables HH				
No subsidy	96.74	89.91	81.25	94.6
Yes subsidy	3.26	10.09	18.75	5.4
Nutrition preg women				
No Nutrition	54.19	60.28	53.91	55.71
Yes Nutrition	45.81	39.72	46.09	44.29
Other benefits				
No benefit	59.53	58.87	87.5	60.2
Yes benefit	40.47	41.13	12.5	39.8

As is evident from the discussion, government efforts led to a positive outcome. Households that benefited from government programs experienced less anxiety. Since the situation was so undesirable, even very little action had an impact.

## Discussion and conclusion

The COVID-19 pandemic triggered several emotional reactions, including fear, worry, and concern. According to a Young Lives telephone survey, 89% of respondents said they were nervous about the COVID-19 situation. There were, however, differences in the effects of the pandemic between socioeconomic groups. We investigate whether COVID-19 made a certain socioeconomic group more vulnerable as a result of the pandemic. For instance, in India, women are often oppressed and have more domestic responsibilities. It is therefore relevant to investigate whether gender bias increased after COVID-19 and if women were more burdened with household chores. The income inequality in India is also high. This is particularly noticeable in states such as Andhra Pradesh and Telangana. We examined how effective financial health and material possessions were in protecting individuals during this difficult time. Additionally, we examined whether people of a particular age group were more affected than others.

For this purpose, an individual's anxiety level was divided into three categories: low, medium, and high. The likelihood of falling into a particular category and experiencing a certain level of anxiety was examined using regression analysis, taking into account many factors, such as gender, job loss, the death of a family member, and food shortages.

The key results from the analysis suggest that on average, women experienced higher anxiety levels than men did. The possible reasons for this were found to be the increased burden of household chores and childcare responsibilities during the pandemic compared with men. About 63% of women reported spending more time doing household chores, and 43% reported spending more time on childcare due to the pandemic. Also, women were laid off at a higher rate than men because of the pandemic: About 71% of women as opposed to 65% of men reported that they were laid off during the pandemic. This was also due to the segregation of occupation on the basis of gender. These findings are in agreement with other studies on the issue. For instance, in their studies [14, 17] and [42], found that women spent more time caring for their families during the lockdown than men did. In addition [7, 13], and [32] reported an increase in gender-based violence during the lockdown.

A couple of other studies reported a decline in the number of women employed, which they attributed to job segregation; a huge layoff in the informal sector, where the majority of women work; and the return of men to villages due to the lockdown. These are the studies by [3, 4, 7, 15, 16] and [42]. According to several studies, including [7] and [23], women’s food intake was also reduced, and their sanitary and hygienic needs were compromised as a consequence of reduced income due to COVID-19.

Many households, especially the poor, faced a higher level of anxiety because of COVID-19 due to a variety of factors, including job losses and the cessation of many beneficiary schemes. In addition, poor children suffered more than others due to a lack of resources. This led to an increase in their stress and the rise of inequalities in society. Several research studies have validated these findings.

A few studies, [2] and [11] reported that as the poor had to be physically present for the job, they experienced higher job losses than the computer-dependent workers. Even those who had jobs had to do so in extremely precarious conditions. Because of these reasons, their anxiety level was highest. The results of this study are similar to those [12, 28], and [35]. These studies revealed that job losses were most severe among the poor. A study on Bangladesh by [35] found that 57% of people went outside daily for work. A few studies like [9], have also found that the poor had limited opportunities and had to work in unsafe conditions for their livelihoods, thus increasing their health risks.

Several studies, such as [5, 31, 40, 41], and [43] also reported that the cessation of schemes to provide meals to children due to school closures magnified the food insecurity problem of low-income populations, especially in low-income countries. Some studies like [1] and Gupta and [21] reported a rise in child abuse and child neglect. Furthermore, as [22] noted, the lack of access to the information technology infrastructure and devices led to a more adverse academic impact on children belonging to vulnerable categories. This is likely to jeopardize their future earning capabilities and thus might increase income inequalities [40].

The downward trend in income was consistent across all income levels. Sixty-two per cent of those who identified themselves as rich now identified themselves as "comfortable" income earners. And higher anxiety was associated with greater material deprivation.

Furthermore, research suggests that the rich were also stressed because of the Covid related factors. The high level of engagement at home was the primary reason for this. The relatively low labor costs in India made it possible for rich people to hire and outsource their basic household chores before COVID-19. However, due to the associated protocols, domestic help was no longer available during COVID-19. According to the regression results, the probability that a rich household compared with a comfortable household would experience high anxiety was 12% higher for those involved in childcare, 22% higher for those experiencing job loss, and 13% higher for those participating in household chores.

Additionally, age was a significant factor in determining the depth of Covid-19's impact on mental health. According to the results, the younger cohort reported high levels of anxiety, which could have stemmed from a variety of factors. Due to the closure of schools and the lack of mobile phones or laptops, many of them had to discontinue their studies. The lack of physical interaction also increased health risks for children. School closure not only affected children’s learning but also isolated them socially. A few students furthermore reported deteriorating relationships with their parents, which contributed to their anxiety.

Similar concerns were highlighted in other studies. In terms of the differences in the impacts of the lockdowns, based on age, [8, 21, 37, 41] and [43] reported various health issues among children, such as increasing weight, a lack of vitamin D, and back pain.

In addition to the young, the elderly were also affected. About 10% of elderly individuals reported high anxiety. The results are consistent with other studies. Few studies, like [6, 30], and [34] reported higher anxiety levels among the elderly, as they were most susceptible to the infection and more likely to be severely affected. Many of them reported that all of their savings were wiped out during the lockdown, and those not receiving pensions had to face greater hardship [29] and [34].

COVID-19 has had a profound impact on multiple dimensions of individuals of diverse backgrounds, which is reflected in their mental health. In light of the importance of mental health in overall wellness, these findings should be taken seriously and should be used to identify potential challenges. A careful mechanism should be laid down to address these challenges in the future.

The paper contributes by combining all the effects of COVID-19 into a single measure, which, though not, strictly speaking, may be used to compare individuals from different socioeconomic groups to identify the characteristics of the person who experienced the most adverse effects of Covid-19. Another major strength of this paper is the large number of questions used to create the index. Consequently, the index is reliable and consistent with existing theory.

The study also has a few limitations. First, the study is based on the states of Andhra Pradesh and Telangana from where the sample is drawn. Furthermore, the sample is largely derived from the original Young Lives sample. The primary objective of the original sample was very different from the purpose of this study. The original sample was pro-poor. It is therefore prudent to generalize the results to other parts of the country and to other sections of society with caution.

In addition, the COVID-19 pandemic was both a national and a state subject. To deal with the crisis, states were given autonomy. As a result, various states handled the pandemic differently. The policy measures discussed here pertain only to the state-specific policies that the Andhra government enforced. At the nationwide level, the situation might have been different in India.

A pandemic like COVID-19 is both a national and state concern. States were given autonomy to deal with the crisis. As a result, different states handled the pandemic differently. In this article, we discuss only state-specific policies enforced by the Andhra government. It is likely that the situation was dealt differently at the national level.

In future work, this index may be used for similar purposes in other countries, as a means of assessing the impact of Covid-19 on individuals. In this context, it is both important and interesting to evaluate if the experiences in one country relate to those in the other and if there are any lessons to be learned.

## Data Availability

The datasets Listening to Young Lives at Work: COVID-19 Phone Survey, First Call and Second Call, 2020, analysed during the current study are available in the repository, [http://doi.org/10.5255/UKDA-SN-8678-3].
